# Adolescent Inpatients With Eating Disorders: Comparison Between Acute and Chronic Malnutrition States on a Refeeding Protocol

**DOI:** 10.1177/2333794X19839780

**Published:** 2019-04-08

**Authors:** Yung-Chieh Chang, Margaret Thew, Kyndal Hettich, Melodee Nugent, Pippa Simpson, M. Susan Jay

**Affiliations:** 1 Hualien Tzu Chi Hospital, Hualien; 2 Medical College of Wisconsin, Milwaukee, WI, USA; 3 Children’s Hospital of Wisconsin, Milwaukee, WI, USA

**Keywords:** refeeding syndrome, eating disorders, anorexia nervosa, inpatient, nutrition, electrolyte imbalance, medical stabilization

## Abstract

This study is a comparison of eating disorder patient outcomes on similar nutrition regimens regardless of degree of malnutrition. Acuity of symptoms in chronically and acutely malnourished patients were analyzed to determine the influence the duration of malnutrition has on refeeding syndrome. Patient outcomes did not differ based on chronicity of malnutrition and are compatible with previous studies in terms of weight gain, medical stabilization, and rate of electrolyte imbalance.

## Introduction

Eating disorders (EDs) are complex biopsychosocial disorders with significant medical sequela and high mortality rate. Onset is usually during adolescence or young adulthood. Adolescents with EDs are best managed by a multidisciplinary team, including a medical provider, therapist, and dietitian.^1^ Weight restoration and nutritional rehabilitation are fundamental components in the treatment of adolescents with anorexia nervosa (AN). In the past, nutritional rehabilitation was based on conservative, consensus-based recommendations due to concerns of refeeding syndrome.^2^ Refeeding syndrome is complex and consists of a variety of metabolic and clinical features, the hallmark being hypophosphatemia. Studies have shown that the degree of hypophosphatemia correlates with the degree of malnutrition, percent median body mass index (%mBMI), and the rate of weight loss before admission.^3,4^ Studies have not shown refeeding hypophosphatemia to be associated with prescribed caloric intake.^5^ Refeeding syndrome occurs more frequently within the first weeks of nutritional rehabilitation; especially inpatients <70% mBMI.^4^ The 2015 position paper from the Society for Adolescent Health and Medicine (SAHM) proposed classification of the degree of malnutrition for adolescents and young adults with EDs.^1^ The components of malnutrition included the following: %mBMI, BMI *z* score, and rate of weight loss. Currently, there is little information about how chronicity and rapidity of weight loss may interact with BMI to affect refeeding syndrome risk.^2^

In addition to risk for developing refeeding syndrome, other indicators for admitting adolescents with EDs include severe bradycardia and orthostatic changes.^1^ Bradycardia is a nearly universal finding in AN, particularly in patients with very low body weight and malnutrition.^6^ Bradycardia has been widely theorized to reflect increased resting vagal tone as an adaptive response to conserve energy in the setting of decreased energy intake.^7-9^ Bradycardia and orthostatic hypotension are expected to resolve slowly over time after initiation of nutritional rehabilitation.

Studies related to the physical responses after refeeding are limited, and no clear markers can be used to predict the outcomes of initial medical stabilization and nutrition. In the present study, subjects were divided into 2 groups based on their duration of weight loss—acute versus chronic malnutrition—to see if it affected refeeding parameters.

## Methods

### Study Design

**Figure 1.**
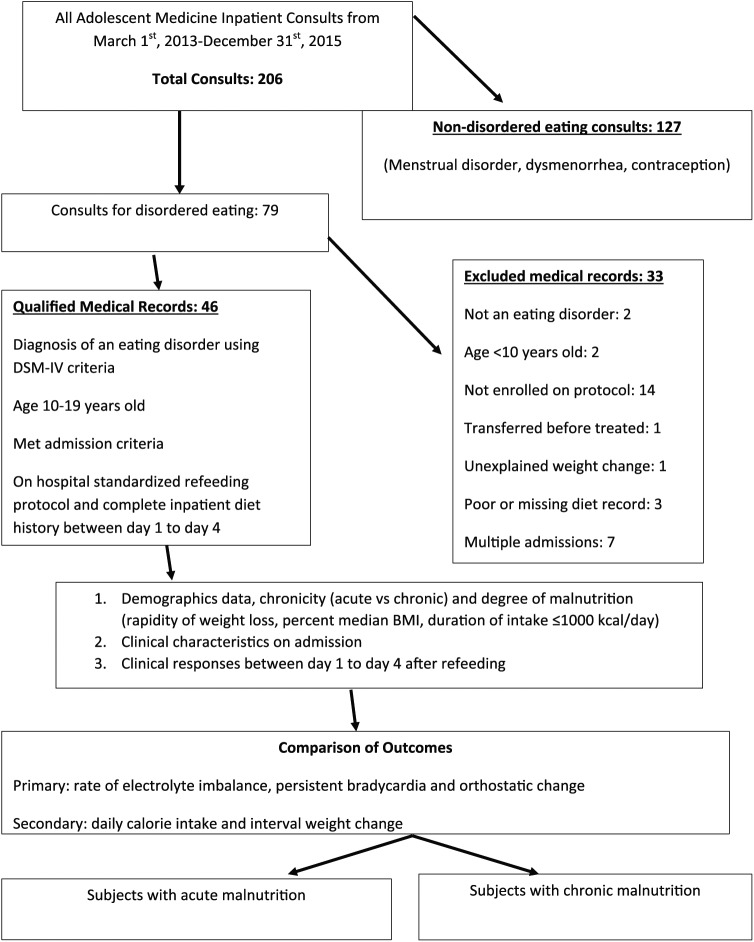
Enrollment criteria.

A retrospective electronic chart review of inpatient consultations to Adolescent Medicine for disordered eating from March 1, 2013 to December 31, 2015 was conducted. A multidisciplinary team including a physician, nurse practitioner, and registered dietitian reviewed charts independently, and there was positive interrater reliability. Eligible subjects were adolescents 10 to 19 years of age, with the diagnosis of an ED confirmed by child psychiatrists within or prior to the admission using the *Diagnostic and Statistical Manual of Mental Disorders, 4th Edition* (*DSM-IV*). Following admission, patients were placed on our standardized refeeding protocol. Patients were assessed and prescribed a nutrition plan by a registered dietitian. Nutrition data between day 1 and day 4 of hospitalization was included, reviewed, and verified. Only charts with a complete nutrition history were included. The flow sheet in Figure 1 demonstrates the enrollment criteria and data collecting strategy in this study. Criteria for admission include severe malnutrition (<75% mBMI), acute weight loss or food refusal, cardiac abnormalities (heart rate <45 beats/min, arrhythmias, hypotension, and orthostatic change), electrolyte imbalance, hypothermia, acute medical complications of malnutrition, and failure of outpatient treatment. Only the first admission was included for subjects with multiple admissions to ensure the response to refeeding would not reflect the previous treatment.

### Ethical Approval and Informed Consent

The Institutional Review Board of the Children’s Hospital of Wisconsin (Approval No. 853814-1, CHW 16/33) waived the need for ethics approval and the need to obtain consent for the collection, analysis, and publication of the retrospectively obtained and anonymized data for this non-interventional study.

### Study Definitions

Physical responses and electrolyte imbalances were assessed following the first day of nutrition if at least one of the following criteria were observed: bradycardia (daily lowest supine heart rate less than 45 beats/min), orthostatic changes (drop in systolic blood pressure >20 mm Hg, drop in diastolic blood pressure >10 mm Hg, or an increase in heart rate >20 beats/min on standing; measured from lying position, sitting for 5 minutes, and standing for 2 minutes), hypothermia (temperature ≤35.5°C), hypokalemia (<3.0 mEq/L), hypophosphatemia (<3.0 mg/dL), hypomagnesemia (<1.8 mg/dL), and hypoglycemia (<60 mg/dL). The rate of refeeding-related electrolyte imbalance was defined as any hypophosphatemia, hypokalemia, or hypomagnesemia after the first day of nutrition. Subjects with initial electrolyte imbalance at admission were categorized as malnutrition related. Subjects were grouped by a modified definition of chronicity in pediatric malnutrition from the American Society of Parenteral and Enteral Nutrition.^10^ Patients with malnutrition for 3 months or less were considered acute, and patients with malnutrition for greater than 3 months were defined as chronic malnutrition.

### Data Collection

Children’s Hospital of Wisconsin Institutional Review Board (IRB) approved data collection protocols for the present study. Data obtained at admission included demographic, anthropometric measures (weight, height, BMI, %mBMI), duration of ED behavior, duration and monthly weight loss percentage, vital signs, orthostatic changes, comprehensive metabolic panel, total daily caloric intake (kcal/kg/day), and phosphate supplement (mg/kg/day). Subjects were grouped by a modified definition of chronicity in pediatric malnutrition from the American Society of Parenteral and Enteral Nutrition.^10^ Patients with malnutrition for 3 months or less were considered acute, and patients with malnutrition for greater than 3 months were defined as chronic. The total percentage of weight loss was calculated by the maximum weight available on the growth chart before admission less the weight at admission; monthly weight loss percentage was defined as the total percentage weight loss divided by the duration of weight loss.

### Inpatient Refeeding Protocol

Our ED inpatient treatment guideline was implemented in 2013; this guideline was adapted and modified based on the protocol from Children’s National Eating Disorder Program located in Washington, DC. The protocol includes the following: orthostatic blood pressure every morning and continuous cardiac monitoring throughout the admission. Patients are weighed daily with 2 gowns, post void, and blinded to weight. Vital signs obtained every 4 hours and laboratory collection every 12 hours include the daily lowest documented supine heart rate, blood pressure, body temperature, and daily potassium, phosphate, and magnesium. Energy needs were determined by the registered dietitian using estimated energy requirement, and structured meal plans including overnight nasogastric tube feeds using a 1.5 cal/mL formula were prescribed for the day. Prescribed calories are increased approximately 250 kcal every 24 hours. Goal calories are met on approximately day 4 of hospitalization. Supplements given for any uneaten food at meals and snacks consist of 65% carbohydrate, 14% protein, and 21% fat. Individual physicians covering the service adjust phosphate supplements based on the available serum phosphate data (potassium phosphate 250-500 mg/tablet up to 4 times a day).

### Statistical Methods

The nonparametric Mann-Whitney test was used to compare continuous variables (Kruskal-Wallis test for more than 2 groups). The continuous variables were the following: age, duration of ED behavior, admission days, %mBMI, BMI value, monthly weight loss percent, weight loss months, daily calorie intake, and phosphate supplement. The Fisher’s exact test was used to compare categorical variables. The categorical variables included gender, disease category, bradycardia, orthostatic change, hypothermia, abnormal alanine aminotransferase (ALT), renal insufficiency, and electrolyte. A *p* value of < .05 was considered statistically significant.

## Results

### Comparison of Acute and Chronic Malnutrition Clinical Features

Those with acute malnutrition had a higher monthly weight loss percent than those with chronic malnutrition, 7.0% versus 2.9%, *P* ≤ .001. The weight loss duration in months including those with acute malnutrition had a lower median weight loss than those with chronic malnutrition, 1.5 months versus 7.5 months, *P* ≤ .001 (Table 1). There were no other significant differences between chronically malnourished versus acutely malnourished patients. The following were not found to be significant: orthostasis, hypothermia, caloric intake, phosphate supplementation, and electrolyte abnormalities (Table 2).

**Table 1.** Comparison of Clinical Characteristics on Admission Between Chronic and Acute Malnutrition.

Clinical Characteristics on Admission (N = 46)	Chronic Malnutrition (N = 21)		Acute Malnutrition (N = 25)		*P*
	N	N (%) or Median (Range)	N	N (%) or Median (Range)	
Monthly weight loss percent	20	2.9 (1-5.7)	24	7.0 (0.02-26.1)	<.001^a^
Weight loss duration, months	20	7.5 (4-12)	24	1.5 (0.5-3.0)	<.001^a^
Percentage of severe malnutrition	21	16 (76)	24	16 (67)	.53
Bradycardia (<45 beats per minute)	21	10 (48)	25	13 (52)	.99

a The subject values differ for both chronic and acute data for weight loss percentages due to missing data.

**Table 2.** Physical Response and Electrolyte Imbalance Compared Between Chronic and Acute Malnutrition.

Physical Responses and Electrolyte Imbalance (N = 46)	Chronic Malnutrition (N = 21)		Acute Malnutrition (N = 25)		*P*
	N	N (%) or Median (Range)	N	N (%) or Median (Range)	
Physical response (admission to day 4)					
Heart rate change (bpm)	21	1 (−29 to 21)	25	6 (−12 to 18)	.14
Weight (kg) change	21	0.7 (−2.3 to 4.4)	25	1.2 (−0.6 to 3.4)	.16
Consistent bradycardia	21	4 (19)	25	6 (24)	.74
Consistent orthostatic change	21	3 (14)	25	7 (28)	.31
Hypokalemia (<3 mEq/L)	21	1 (5)	25	0	.46
Hypophosphatemia (<3 mg/dL)	21	4 (19)	25	2 (8)	.39
Hypomagnesemia (<1.8 mg/dL)	21	3 (14)	25	2 (8)	.65
Refeeding-related electrolyte imbalance	21	6 (29)	25	4 (16)	.48

### Demographics and Clinical Features on Admission

Of the total 79 consults, 46 subjects met study criteria. Electronic medical records were reviewed: 89.1% female, median age 15.3 years (range 10.1-18.8), 69.6% diagnosed using *DSM-IV* criteria with AN, restrictive type. Median BMI was 15.8 kg/m^2^ (range 11.7-21.7), %mBMI was 80.6% (range 56.7% to 115.8), median total percentage weight loss was 13.6% (range 3.6% to 45.6%), median monthly weight loss was 5% (range 0.02% to 26.1%), and median weight loss duration was 3 months (range 0.5-12). Clinical features at admission included the following: bradycardia 50%, hypothermia 2.2%, orthostatic changes 54.5%, elevated ALT 36.4%, renal insufficiency 27%, hypokalemia 2.5%, hypophosphatemia 2.4%, and hypoglycemia 8.1%. The lowest level of serum potassium and phosphate were 2.2 mEq/L and 2.8 mg/dL, respectively.

The rates of refeeding-related hypokalemia, hypophosphatemia, and hypomagnesemia were 2.2%, 13%, and 10.9%, respectively. Overall refeeding-related electrolyte imbalance was 21.7%. Caloric intake on admission was 800 (200-2100) kcal/day. Planned caloric intake on day 1 was 1935 (815-2625) kcal/day. Dietitian prescribed meal plans between admission to day 4 calculated to 22.6 (4.7-50.1), 47.1 (15-90), 53.6 (33.5-99.8), 56.3 (37.2-107.4), and 59 (27.1-104.9) calories per kilogram per day, respectively (Table 3).

**Table 3.** Comparison of Total Daily Calorie Intake Between 2 Groups^a^.

Total Daily Calorie Intake and Phosphate Supplement (N = 46)	Chronic Malnutrition (N = 21)		Acute Malnutrition (N = 25)		*P*
	N	Median (Range)	N	Median (Range)	
Total calorie (kcal/kg/day)					
Admission	19	22.5 (8.1-50.1)	22	25.5 (4.7-43.4)	.93
Day 1	21	49.1 (15-90.9)	25	40.7 (15.1-61)	.12
Day 2	21	54.6 (34.3-99.8)	25	52.9 (33.5-70.7)	.38
Day 3	21	57.5 (37.4-107.4)	25	55.4 (37.2-72.6)	.24
Day 4	21	60.3 (40.8-104.9)	25	58.3 (27.1-72.8)	.72

a The subject values differ from admission to subsequent days due to missing date from admission for chronic and acute cases.

## Discussion

To our knowledge, this is the first published comparison of chronicity among adolescents with EDs. We provide a comparison of clinical characteristics before and after nutritional rehabilitation between acute and chronic malnutrition. When comparing the components of malnutrition between groups, %mBMI did not show significant differences, but the chronicity and rapidity of weight loss was significant. Not surprisingly, the subjects in the chronic group have more features related to severe malnutrition, such as longer duration of weight loss and more overall weight loss percent but less rapidity of weight loss. Our study did not denote physical findings on admission to be statistically significant indicators of starvation classification (acute vs chronic) including the following: rate of electrolyte imbalance, bradycardia, hypothermia, orthostatic change, abnormal ALT, and renal insufficiency. In addition, after initiation of nutrition, there were no statistically significant changes in heart rate, weight gain, bradycardia, orthostatic changes, or electrolyte imbalance between groups. These findings suggest possible underlying physiological mechanisms in response to starvation and initiation of nutrition, such as bradycardia, which reflects increased resting vagal tone as the body tries to conserve energy.^6^

Limitations in this study include the retrospective study design, subject initial self-report of nutrition intake, short length of stay, and small study population. Additionally, data related to weight loss duration and total percentage of weight loss was obtained through chart review. Despite these limitations, our outcomes are supported by other studies in terms of weight gain, medical stabilization, and rate of electrolyte imbalance.

Refeeding syndrome can be a dangerous and life-threatening response to nutritional rehabilitation of malnourished patients. Clinical presentation of weight changes regarding monthly weight loss and the duration of weight loss were significantly different among subjects. Despite these differences, there was no difference in daily total caloric intake or body weight changes between groups throughout their admission. Our study revealed that chronic malnutrition defined as >3 months did not result in more refeeding syndrome complications. This study supports that chronicity and rapidity of weight loss are not good indicators in predicting the outcomes of nutritional rehabilitation based on length of illness.

Our study was an initial effort to explore the role of chronicity and weight loss as indicators of risk for refeeding syndrome. Similar nutrition prescriptions did not affect markers negatively in this sample population. Both acute and chronically malnourished patients did not demonstrate differences in refeeding laboratory tests and physical response. This is reassuring to providers to prescribe higher rates of nutrition despite differences in length of malnutrition. Future larger prospective studies in EDs will be important to continue the research in refeeding syndrome and the difference between acute and chronic malnutrition.

## References

[1] Society for Adolescent Health and Medicine; Golden NH Katzman DK et al Position paper of the Society for Adolescent Health and Medicine: medical management of restrictive eating disorders in adolescents and young adults. J Adolesc Health. 2015;56:121-125. 25530605 10.1016/j.jadohealth.2014.10.259

[2] Society for Adolescent Health and Medicine. Refeeding hypophosphatemia in hospitalized adolescents with anorexia nervosa: a position statement of the Society for Adolescent Health and Medicine. J Adolesc Health. 2014;55:455-457. 25151056 10.1016/j.jadohealth.2014.06.010 PMC6159900

[3] Garber AK Michihata N Hetnal K Shafer MA Moscicki AB. A prospective examination of weight gain in hospitalized adolescents with anorexia nervosa on a recommended refeeding protocol. J Adolesc Health. 2012;50:24-29. 22188830 10.1016/j.jadohealth.2011.06.011 PMC4467563

[4] Ornstein RM Golden NH Jacobson MS Shenker IR. Hypophosphatemia during nutritional rehabilitation in anorexia nervosa: implications for refeeding and monitoring. J Adolesc Health. 2003;32:83-88. 12507806 10.1016/s1054-139x(02)00456-1

[5] Golden NH Keane-Miller C Sainani KL Kapphahn CJ. Higher caloric intake in hospitalized adolescents with anorexia nervosa is associated with reduced length of stay and no increased rate of refeeding syndrome. J Adolesc Health. 2013;53:573-578. 23830088 10.1016/j.jadohealth.2013.05.014

[6] Sachs KV Harnke B Mehler PS Krantz MJ. Cardiovascular complications of anorexia nervosa: a systematic review. Int J Eat Disord. 2016;49:238-248. 26710932 10.1002/eat.22481

[7] Casiero D Frishman WH. Cardiovascular complications of eating disorders. Cardiol Rev. 2006;14:227-231. 16924163 10.1097/01.crd.0000216745.96062.7c

[8] Galetta F Franzoni F Cupisti A Belliti D Prattichizzo F Rolla M. QT interval dispersion in young women with anorexia nervosa. J Pediatr. 2002;140:456-460. 12006961 10.1067/mpd.2002.122726

[9] Romano C Chinali M Pasanisi F et al Reduced hemodynamic load and cardiac hypotrophy in patients with anorexia nervosa. Am J Clin Nutr. 2003;77:308-312. 12540387 10.1093/ajcn/77.2.308

[10] Mehta NM Corkins MR Lyman B et al Defining pediatric malnutrition: a paradigm shift toward etiology-related definitions. JPEN J Parenter Enteral Nutr. 2013;37:460-481. 23528324 10.1177/0148607113479972

